# In Chaotropy Lies Opportunity

**DOI:** 10.3389/fmicb.2015.01505

**Published:** 2016-01-05

**Authors:** Mark A. Lever

**Affiliations:** Department of Environmental Systems Sciences, Institute of Biogeochemistry and Pollutant Dynamics, Swiss Federal Institute of Technology ZurichZurich, Switzerland

**Keywords:** chaotropic, kosmotropic, chaophilic, chaotolerant, extremophile, fungi, biotic fringe, astrobiology

The known distribution of microbial life on Earth is expanding thanks to improvements in technologies that enable contamination-free sampling of previously inaccessible environments, and the detection and cultivation of microbes from these locations (Lever et al., 2013; Priscu et al., 2013; Šantl-Temkiv et al., 2013; Inagaki et al., 2015). As novel Bacteria, Archaea, and microbial eukaryotes are discovered, and even familiar organisms reveal unknown adaptations to extreme temperature, pressure, radiation, pH, chemical toxicity, desiccation, or osmotic stress, notions of habitability have to be revised. In many cases the “biotic fringe,” i.e., the boundary separating environments that sustain life from environments believed to exclusively host abiotic processes (Shock, 2000), has to be adjusted to include new places that were previously considered devoid of life.

Parallel to knowledge on the distribution of life, the understanding of mechanisms determining where life can exist is transforming. Sustenance of life in any given locale requires that organisms conserve sufficient power to repair the inevitable biomolecule damage that is induced by their environment over time. This basal power requirement (BPR; Hoehler and Jørgensen, 2013) likely varies under different physicochemical conditions. For instance, rates of biomolecule damage on the monomeric (e.g., racemization, depurination) to macromolecular level (e.g., hydrolysis, changes to secondary structure) are a function of temperature. Despite adaptations that result in increased heat stability of their cellular building blocks, organisms adapted to hot environments have to spend orders of magnitude more power on the repair of biomolecules from temperature-related damage than their counterparts living at moderate temperatures (Lever et al., 2015). Comparable increases in BPR likely also result under other physiological extremes, e.g., high pressure, radiation, desiccation, pH, osmotic stress, chemical toxicity, and combinations thereof.

In recent decades, the understanding of how chemical toxicity affects organisms has become more nuanced. While certain toxins alter biomolecules on an intramolecular level, e.g., through oxidation or covalent bonding, others modify the macromolecular structure. In the latter category is a group called “chaotropes” (*Greek* chaos = disorder, tropy = behavior). Chaotropic compounds destabilize the two-dimensional structure and folding patterns of biomolecules by replacing water from the hydration shell and eliminating hydrogen bonds, or by penetrating into hydrophobic parts thereby causing swelling or solubilization of these parts (reviewed in Ball and Hallsworth, 2015). These properties make concentrated solutions with chaotropic compounds, e.g., guanidine chloride or urea, suitable for biochemical and molecular biological applications requiring microbial cell lysis, protein or nucleic acid extraction, and/or denaturation. At sublethal concentrations, the damaging effects of chaotropes on biomolecules result in higher rates of biomolecule repair and increased synthesis and accumulation of compounds that stabilize biomolecules and thereby offset the negative effects of chaotropes—socalled “kosmotropes.” In nature, the maximum concentration of chaotropes, e.g., MgCl_2_ or CaCl_2_, that is tolerated by living organisms is thus to some extent linked to the concentration of compatible kosmotropes, e.g., NaCl (Oren, 1983).

Besides inducing stress, chaotropic compounds also have important beneficial effects on microorganisms. Psychrophilic fungi and fungi in NaCl-rich environments produce the chaotropic compound glycerol to increase biomolecule flexibility at low temperature and to shield enzymes from the damaging kosmotrope Na^+^, respectively, (Albertyn et al., 1994; Chin et al., 2010). Fungi inhabiting brines in sea ice and dry soils may also produce chaotropic sugars and sugar alcohols as compatible solutes to concentrated kosmotropes (Gunde-Cimerman et al., 2003; Rummel et al., 2014). Research on the Dead Sea, pioneered by Benjamin Elazari Volcani 80 years ago (Wilkansky, 1936), has shown that blooms of the dominant phytoplankton (*Dunaliella* spp.) occur only after temporary salinity decreases caused by winter floods (Oren, 1993). *Dunaliella* blooms typically overlap with blooms of halophilic Archaea, in which the chaotrope glycerol, produced by *Dunaliella* as an osmo-protectant, serves as a key energy substrate for Archaea (Oren, 1995). Environments with high salinities, low moisture content, or temperatures below the freezing point of water cover vast areas on Earth—and are widely inhabited by microorganisms. This suggests an important role for adaptations involving the tolerance to, or targeted synthesis of, chaotropic compounds. Yet, the role of chaotropic compounds in limiting or expanding the limits of life on Earth is poorly understood.

Zajc et al. (2014) contribute significantly to knowledge on the chaotropy limits of life by demonstrating for the first time that microbial fungi can tolerate high concentrations of the widespread chaotropes MgCl_2_ and CaCl_2_ in the absence of high concentrations of protective kosmotropes. The authors grew a total of 135 fungal strains with known halo- or xerotolerance, that had been isolated from bitterns (MgCl_2_-rich brines) or the Dead Sea, or obtained from culture collections, at different concentrations of MgCl_2_ and CaCl_2_ in the laboratory. Several fungal strains grew at MgCl_2_ and CaCl_2_ concentrations of 2M in the absence of kosmotropes—a drastic extension of the previous known limit of 1.26M, which was for prokaryotes (Hallsworth et al., 2007). The fact that certain strains tolerated high concentrations of MgCl_2_ but not CaCl_2_, whereas others tolerated high concentrations of both salts, provokes the question of whether or not organisms have specifically evolved in response to chaotrope concentrations in the environment. So-called “chaophiles,” whose existence was first speculated by Hallsworth et al. (2007), or “chaotolerant”(this study) organisms might represent dominant organisms in environments where chaotropic stress is a frequent or permanent phenomenon, such as bitterns, salt deposits, surface, subterranean and submarine lakes, and brines. Future studies on the chaotropy limits in nature, combined with investigations on the effects of sublethal concentrations of chaotropes on microbial power requirements, will bring to light to what extent chalophily or chaotolerance influence and explain the distribution of life on Earth and elsewhere in the Universe.

## Conflict of interest statement

The author declares that the research was conducted in the absence of any commercial or financial relationships that could be construed as a potential conflict of interest.
